# Beta Defensin-2 Is Reduced in Central but Not in Distal Airways of Smoker COPD Patients

**DOI:** 10.1371/journal.pone.0033601

**Published:** 2012-03-16

**Authors:** Elisabetta Pace, Maria Ferraro, Marta Ida Minervini, Patrizio Vitulo, Loredana Pipitone, Giuseppina Chiappara, Liboria Siena, Angela Marina Montalbano, Malcolm Johnson, Mark Gjomarkaj

**Affiliations:** 1 Institute of Biomedicine and Molecular Immunology, National Research Council, Palermo, Italy; 2 Department of Pathology, University of Pittsburgh, Pittsburgh, Pennsylvania, United States of America; 3 Mediterranean Institute for Transplantation and for High Specialised Therapies, ISMETT, Palermo, Italy; 4 GlaxoSmithKline Research and Development Ltd., Uxbridge, Middlesex, United Kingdom; University of Tübingen, Germany

## Abstract

**Background:**

Altered pulmonary defenses in chronic obstructive pulmonary disease (COPD) may promote distal airways bacterial colonization. The expression/activation of Toll Like receptors (TLR) and beta 2 defensin (HBD2) release by epithelial cells crucially affect pulmonary defence mechanisms.

**Methods:**

The epithelial expression of TLR4 and of HBD2 was assessed in surgical specimens from current smokers COPD (s-COPD; n = 17), ex-smokers COPD (ex-s-COPD; n = 8), smokers without COPD (S; n = 12), and from non-smoker non-COPD subjects (C; n = 13).

**Results:**

In distal airways, s-COPD highly expressed TLR4 and HBD2. In central airways, S and s-COPD showed increased TLR4 expression. Lower HBD2 expression was observed in central airways of s-COPD when compared to S and to ex-s-COPD. s-COPD had a reduced HBD2 gene expression as demonstrated by real-time PCR on micro-dissected bronchial epithelial cells. Furthermore, HBD2 expression positively correlated with FEV1/FVC ratio and inversely correlated with the cigarette smoke exposure. In a bronchial epithelial cell line (16 HBE) IL-1β significantly induced the HBD2 mRNA expression and cigarette smoke extracts significantly counteracted this IL-1 mediated effect reducing both the activation of NFkB pathway and the interaction between NFkB and HBD2 promoter.

**Conclusions:**

This study provides new insights on the possible mechanisms involved in the alteration of innate immunity mechanisms in COPD.

## Introduction

Chronic obstructive pulmonary disease (COPD) is an increasingly serious global health problem [1] and it is expected to be the third most common cause of death in 2020 [2].

Distal airway bacterial colonization may occur in COPD patients, who often have altered pulmonary defenses [3]. A key component of the innate defences against infections is represented by the toll like receptor (TLR) family [4]. Upon activation of TLR by endogenous and exogenous ligands, the release of chemokines including IL8 and IP-10 and of defensins may occur [5]. TLR2 and TLR4, predominantly expressed by monocytes/macrophages and neutrophils [4], are also expressed by lung and bronchial epithelial cells [6]. The airway epithelium is active in airway defence mechanisms releasing cytoprotective mucus and defensins [7] and plays an important role in coordinating local inflammation and immune responses through the generation of cytokines and chemokines [8].

The tobacco smoking habit interferes with the innate host defense system by increasing mucus production, reducing mucociliary clearance, reducing human beta 2 defensin (HBD2) release [9], disrupting the epithelial barrier and stimulating the migration of inflammatory cells into the damaged tissue [10].

Although it is known that cigarette smoke exposure, a major determinant of COPD, is able to alter the expression and the activation of TLR4 in a bronchial epithelial cell line [11], it is unknown whether this phenomenon occurs in vivo and whether it is differently altered at different levels of the bronchial tree. In COPD, the predominant pathology is present in peripheral airways and lung parenchyma [12]. To what extent central airways may mirror events occurring in distal lung is uncertain.

The aim of the present study was to evaluate whether COPD is associated to the alteration of the expression of TLR4 or to an altered expression of human beta 2 defensin (HBD2) in central as well as in distal airways.

## Materials and Methods

### Patient Population

Patients underwent surgery for lung cancer and were recruited at ISMETT-Palermo, Italy. The study was approved by the ISMETT Ethic Committee (#149311-29/05/2006) and was in agreement with Helsinki Declaration. Written informed consent was obtained from each patient. The following patient groups were selected: 1) never smoking patients without COPD (C) (n = 13); 2) smoking patients (>15 packs/year) without COPD (S) (n = 12); 3) smoking patients (>15 packs/year) with COPD (s-COPD) (n = 17); 4) ex smoker patients (>15 pack/year) who had stopped to smoke by more than one year and with COPD (ex-s-COPD) (n = 8). COPD patients were treated with bronchodilators and were classified on the basis of preoperative lung function: FEV_1_ less than 80% of reference, FEV_1_/FVC less than 70%, and bronchodilatation effect less than 12%. The patients were not under corticosteroid therapy (neither inhaled nor systemic) and not under antibiotics and did not have exacerbations during the month preceding the study. Subjects had negative skin tests for common allergen extracts and had no past history of asthma or allergic rhinitis.

### Immunohistochemistry

Tissue specimens from tumor-free central bronchi and peripheral lung tissue were selected, fixed with 10% Neutral buffer formalin and embedded in paraffin wax. Sequential sections (3 µm thick) were placed on poly-L-lysine coated slides, deparaffinized in xylene, rehydrated in a descending ethanol series and stained with haematoxylin and eosin (HE).

Immunohistochemistry and image analysis were used to determine TLR4, and HBD2 expression using rabbit polyclonal antibodies (Santa Cruz Biotechnology, Santa Cruz, CA) in central (internal perimeter >6 mm) and distal (internal perimeter < or  = 6 mm) airways [13]. LSAB2 Dako kit (Code N° K0674) (Dako, Glostrup, Denmark) and Fuchsin Substrate-Chromogen System Dako [14] were used for the staining. Rabbit negative control immunoglobulins (Dako) were used for negative controls. The immunoreactivity was evaluated blindly by 2 independent investigators using a Leica (Wetzlar, Germany) microscope ×400 magnification. The length of the basement membrane was evaluated using a Quantimet 500 MC software (Leica) for Image Analysis. Results were expressed as the number of positive epithelial cells/mm basement membrane as reported in a similar COPD study [15].

### Laser capture microdissection

Laser capture microdissection (LMD) was performed using the Leica AS LMD (Leica Microsystems, Germany) [16] from three s-COPD and three ex-s-COPD. Epithelial cells (recognized by morphologic characteristics) were microdissected from the sample into the cap of a microtube and then processed in the same tube. Further details are provided in the online supplement.

### Real time PCR

Real time PCR was performed as previously described [17]. Total cellular RNA was extracted from s-COPD and ex-s-COPD micro-dissected tissues using RNeasy Microkit (Qiagen, Milan, Italy) and reverse-transcribed to cDNA, using Superscript First-Strand Synthesis System for RT-PCR (Invitrogen, Carlsbad, CA, USA). Real-time quantitative PCR of HBD2 gene was carried out on ABI PRISM 7900 HT Sequence Detection Systems (Applied Biosystems, Foster City, CA, USA) using specific FAM-labeled probe and primers (Applied Biosystems, TaqMan Assays on Demand). GAPDH gene expression was used as endogenous control for normalization. Relative quantification of mRNA was carried out with comparative CT method.

### Stimulation of bronchial epithelial cell lines

16-HBE, an immortalized normal bronchial epithelial cell line, was used in this study [18].

16HBE were cultured with or without IL-1β (30 ng/ml) (R&D System, Minneapolis, MN) and with or without 10% cigarette smoke extracts (CSE) for 24 hrs as previously described [11]. At the end of stimulation cell extracts were collected for assessing HBD2 m-RNA expression by Real Time PCR and for assessing HBD2 protein by flow cytometry, IkB protein expression by western blot analysis and for ChiP analysis.

### Flow cytometry

For flow cytometry, analyses were performed on a Becton Dickinson FACSCalibur System using a rabbit polyclonal antibody anti-HBD2 (Santa Cruz Biotechnology) followed by a fluorescein isothio-cyanate (FITC) conjugated anti-rabbit IgG (Dako).

Analysis was done on 100,000 acquired events for each sample using cellQuest acquisition and data analysis software (Becton Dickinson (BD) Mountain View, CA). Negative controls were performed using an isotype control antibody (BD PharMingen, Mountain View, CA). For the detection of intracellular HBD2, 16-HBE were cultured overnight with GolgiStop (2 µM final concentration) (BD PharMingen). Cells, washed twice in PBS with 1% FCS, were fixed with PBS containing 4% paraformaldehyde for 20 min at room temperature. After two washes in permeabilization buffer (PBS containing 1% FCS, 0.3% saponin, and 0.1% Na azide) for 15 min at 4°C, the cells were stained with rabbit polyclonal antibody anti-HBD2 (Santa Cruz Biotechnology) followed by a fluorescein isothio-cyanate (FITC) conjugated anti-rabbit IgG (Dako)and then evaluated by flow-cytometry.

### Western blot analysis

The expression of phosphorylated IkB alpha (p IkBa) was evaluated by western blot analysis as previously described [11]. 40 µg of total protein were loaded in the gel. All blots were first probed using a rabbit polyclonal antibody anti-pIkBa (1∶500) (Cell Signaling Technology Inc) and a rabbit polyclonal antibody anti-IkBa (1∶1000) (Cell Signaling Technology Inc). Revelation was performed with an enhanced chemioluminescence system (GE Healthcare, Chalfont St. Giles, UK) followed by autoradiography. Beta-actin (Sigma) was used as housekeeping protein to normalize differences in protein loading.

### ChiP Analysis

ChiP analysis was performed using the EZ-ChIP kit (Upstate-Millipore Corporate- Billerica, MA) as previously described [19].

The 16-HBE were stimulated as above mentioned and the crosslinked chromatin were sonicated to lengths spanning 200–1000 bp. The samples were precleared with 60 µl of Protein A Agarose and then incubated with a rabbit polyclonal antibody anti human NFkB (Santa Cruz Biotechnology). Immunocomplexes were precipitated using Protein A Agarose. After washing, DNA fragments were isolated and purified with columns. PCR was performed using primers spanning the promoter region of HBD2 gene using the primers: sense 5′-catcccccagtctcttcatct-3′ and antisense 5′-atgagaccagtgtccaggcta-3′; sense 5′-ggtgtgaatggaaggaactca-3′ and antisense 5′-ttcagctcctggggatgatac-3′; sense 5′-tggcaggttataggtcctgag-3′ and antisense 5′-ataaaggtcctggtccctggt-3′ [20].

### Statistics

Age, clinical scores of the patients and Real time PCR data are expressed as mean± standard deviation. The Kruskal Wallis and Mann Whitney U-test were used for comparisons between patient groups. The Spearman test was used for correlations. Student's paired t test was used for in vitro experiments on 16HBE. P<0.05 was accepted as statistically significant.

## Results

### Demographic characteristics of the subjects

The demographic characteristics and the functional evaluations of the studied groups are shown in Table 1. All recruited patient groups were similar with regard to age.

**Table 1:** Demographic characteristics of the subjects.

	Controls = 13	Smokers = 12	s-COPD = 17	ex-s-COPD = 8
Gender (M/F)	9/4	10/2	15/2	7/1
Age (years)Means±SD	60±13	63±9	64±8	70±7
Packs/year	-	59±21	70±31*	40±24
FEV1 % of predictedMeans±SD	89±16	77±12	68±15	78±8
FEV1/FVC% of predictedMeans Means±SD	83±6	80±9	60±7	65±4

* p<0.01 vs ex-COPD.

The number of packs/year was similar between S and s-COPD but it was significantly higher in s-COPD than in ex-s-COPD (p<0.01).

### TLR4 expression

**Figure 1:**
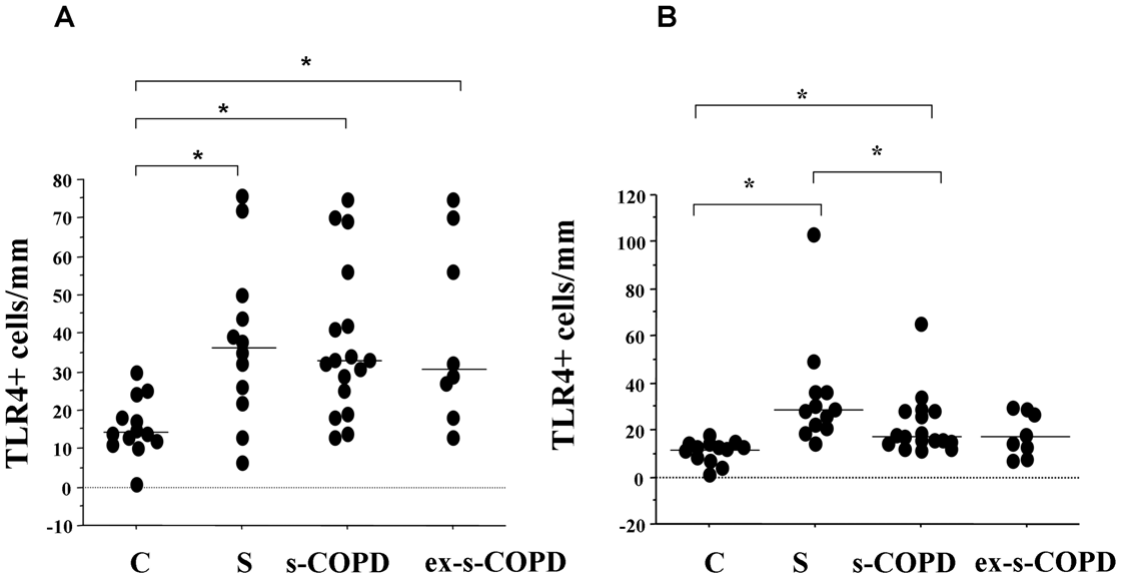
Expression of TLR4 in distal and in central airways. Immunohistochemistry for TLR4 in distal (A) and in central airways (B) from surgical samples of Controls (n = 13), S (n = 12), s-COPD (n = 17) and ex-s-COPD (n = 8) subjects. Cells were stained with an anti-TLR4 antibody. Negative control were performed using rabbit immunoglobulins negative control (see materials and methods for details). **A**) Individual counts for the number of positive epithelial cells/mm basement membrane in distal airways. Horizontal bars represent median values. * p<0.05 values in figure represent Mann-Whitney U test analyses. **B**) Individual counts for the number of positive epithelial cells/mm basement membrane in central airways. Horizontal bars represent median values. * p<0.05 values in figure represent Mann-Whitney U test analyses.

**Figure 2:**
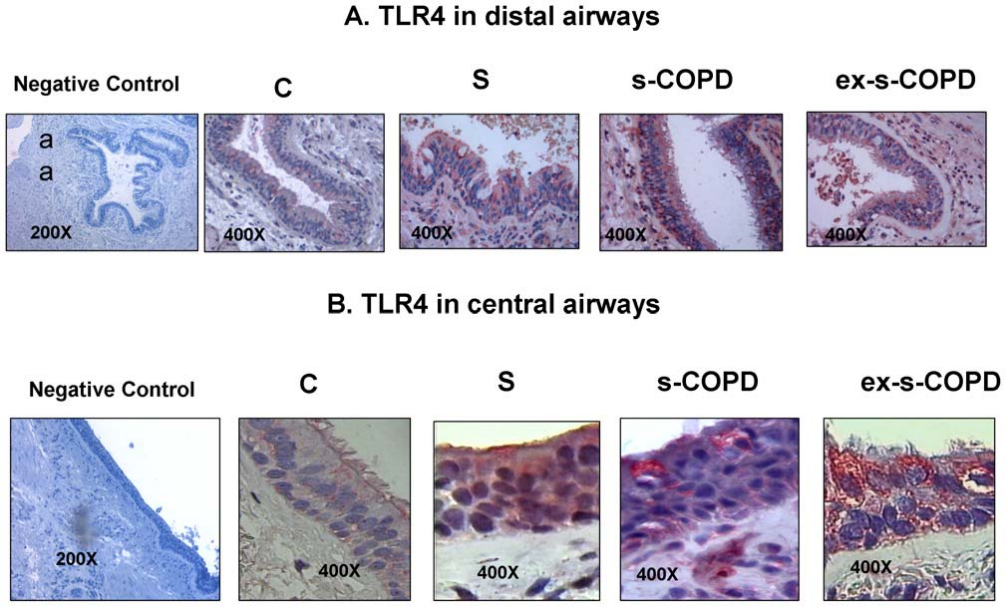
TLR4 immunostaining in distal and in central airways. **A**) Representative negative control and representative TLR4 immunostaining (red stain) in distal airways of a Control, of a Smoker, of a s-COPD and of an ex-s-COPD. **B**) Representative negative control and representative TLR4 immunostaining (red stain) in central airways of a Control, of a Smoker, of a s-COPD and of an ex-s-COPD. For central airways a particular from a 400× magnification was selected and showed.

Since smoking habit interferes with the innate host defense system [10], we first assessed TLR4 expression in airway epithelial cells of distal and of central airways. TLR4 expression was increased in the epithelium of distal airways (figure 1 A and figure 2 A) in S, in s-COPD and in ex-s-COPD when compared to C. In central airways TLR4 expression was increased in S and s-COPD when compared to C and the TLR4 expression was significantly higher in S than in s-COPD (figure 1 B and figure 2 B). TLR4 expression were detected in basal and in columnar epithelial cells.

### HBD2 Expression

**Figure 3:**
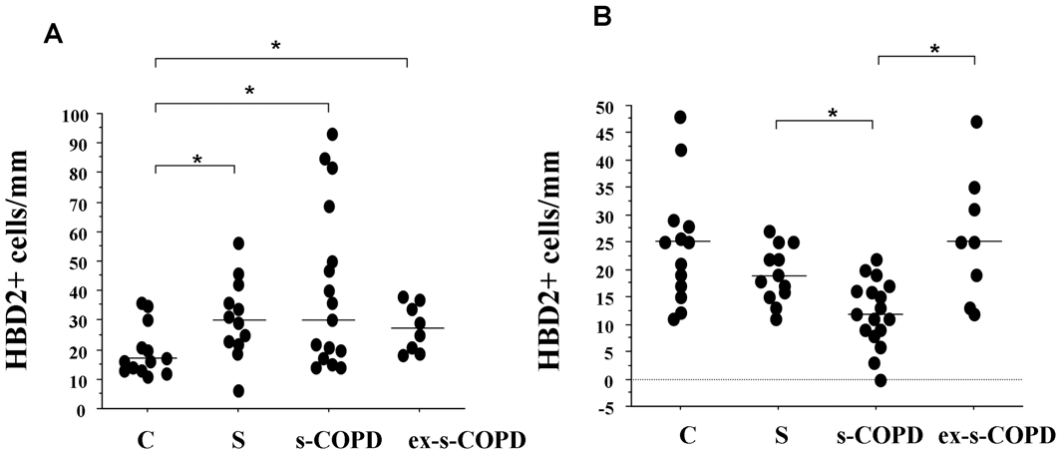
Expression of HBD2 in distal and in central airways. Immunohistochemistry for HBD2 in distal (A) and in central airways (B) from surgical samples of Controls (n = 13), S (n = 12), s-COPD (n = 17) and ex-s-COPD (n = 8) subjects. Cells were stained with an anti-HBD2 antibody. Negative control were performed using rabbit immunoglobulins negative control (see materials and methods for details). **A**) Individual counts for the number of positive epithelial cells/mm basement membrane in distal airways. Horizontal bars represent median values. * p<0.05 values in figure represent Mann-Whitney U test analyses. **B**) Individual counts for the number of positive epithelial cells/mm basement membrane in central airways. Horizontal bars represent median values. * p<0.05 values in figure represent Mann-Whitney U test analyses.

**Figure 4:**
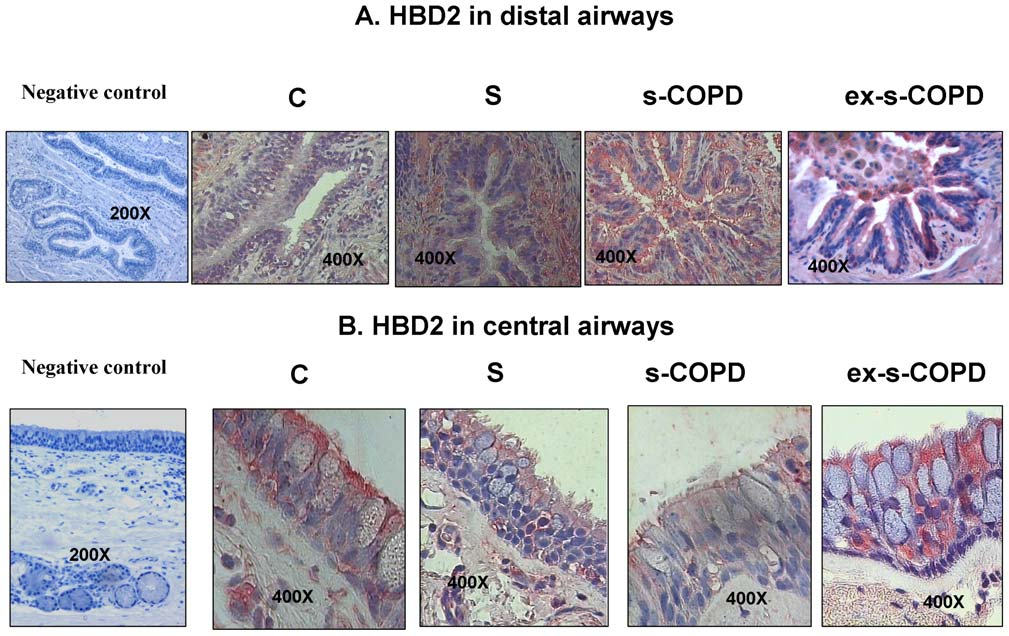
HBD2 immunostaining in distal and in central airways. **A**) Representative negative control and representative HBD2 immunostaining (red stain) in distal airways of a Control, a Smoker, a s-COPD and an ex-s-COPD. **B**) Representative negative control and representative HBD2 immunostaining (red stain) in central airways of a Control, of a Smoker, of a s-COPD and of ex-s-COPD. For central airways a particular from a 400× magnification was selected and showed.

Since the activation of TLR4 leads to the release of defensins [21], HBD2 expression was assessed. In the epithelium of distal airways, HBD2 expression was increased in S, s-COPD and in ex-s-COPD when compared to C (figure 3 A and figure 4 A). In the epithelium of central airways, HBD2 expression was significantly reduced in s-COPD when compared to S and to ex-s-COPD (figure 3 B and figure 4 B).

### Correlations

**Figure 5:**
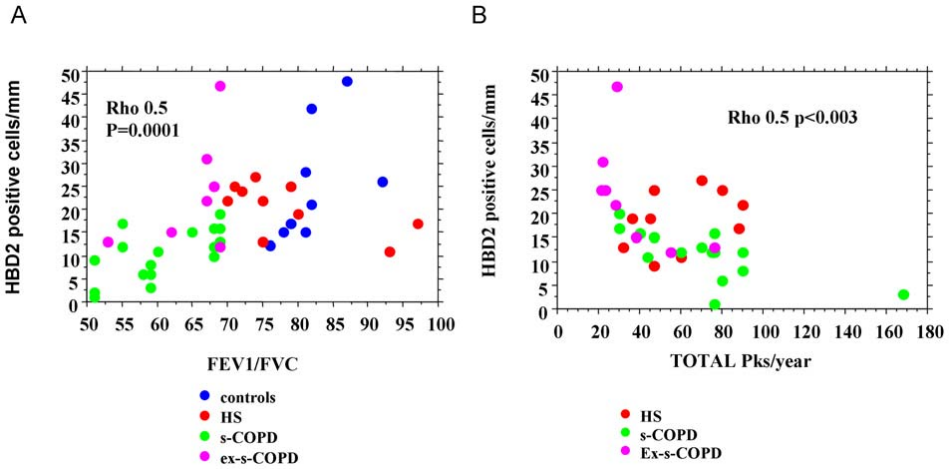
Correlations between the expression of HBD2 in central airways and functional parameters. The expression of HBD2 in central airways of Controls (n = 13), S (n = 12), s-COPD (n = 17) and ex-s-COPD (n = 8) was correlated with FEV1/FVC ratio (**A**) and packs/year (**B**) by Spearman Correlation test.

We assessed whether TLR4 and HBD2 expression, in distal and in central airways, correlated with FEV1 and with FEV1/FVC ratio or with cigarette smoke exposure (packs/year). No significant correlation was found between these parameters and TLR4 expression (data not shown). A significant correlation was observed between the HBD2 expression in central airways and FEV1/FVC ratio (Figure 5 A) but not between HBD2 and FEV1 (data not shown). Furthermore, HBD2 expression in central airways inversely correlated with cigarette smoke exposure (packs/year) (Figure 5 B).

### HBD2 mRNA expression in microdissected bronchial epithelium

**Figure 6:**
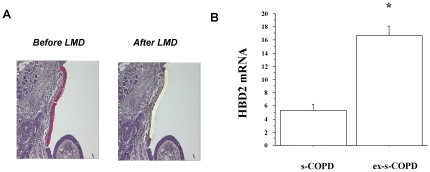
Expression of HBD2 m-RNA in central airways. HBD2 m-RNA expression was assessed by Real time PCR in microdissected bronchial epithelium from s-COPD (n = 3) and from ex-s-COPD (n = 3) (see materials and methods for details). **A**) Representative images showing the bronchial epithelium before (on the left) and after (on the right) laser microdissection (LMD). **B**) Expression of HBD2 m-RNA in microdissected bronchial epithelium. GAPDH gene expression was used as endogenous control for normalization. Relative quantitation of mRNA was carried out with comparative CT method. (mean±SD). * p<0.05.

To understand whether a reduced mRNA expression was responsible of the different HBD2 expression between s-COPD and ex-s-COPD in central airways, real time PCR was performed on microdissected bronchial epithelium (figure 6 A) from the two patient groups. Decreased HBD2 mRNA expression was observed in s-COPD in comparison to ex-s-COPD (figure 6 B).

### In vitro effects of cigarette smoke in bronchial epithelial cells

**Figure 7:**
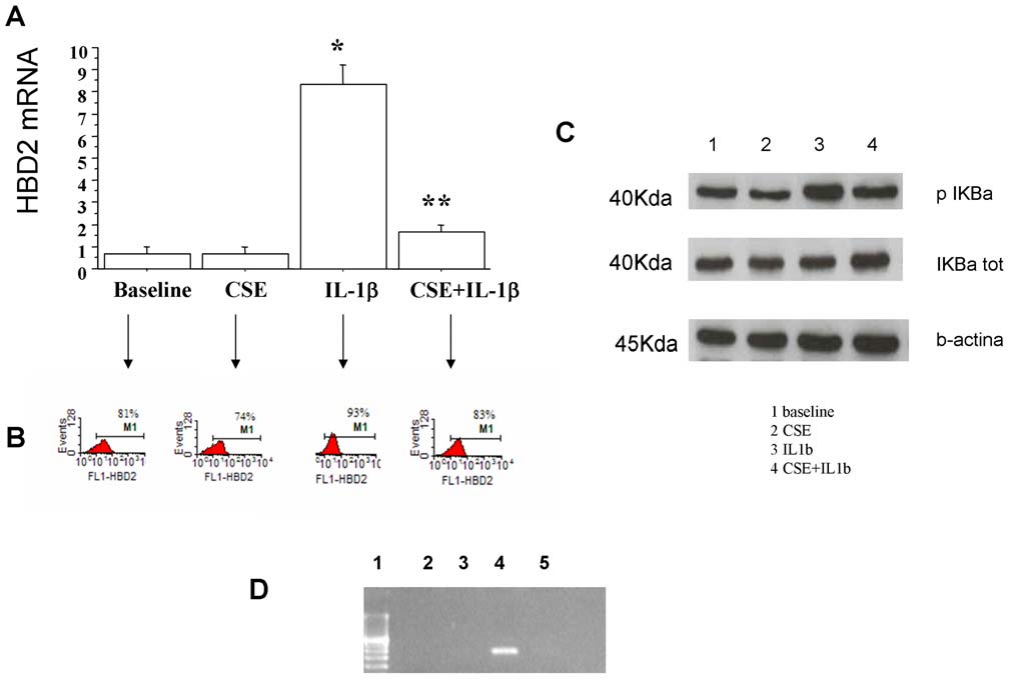
Effects of CSE in bronchial epithelial cells (16-HBE). 16-HBE cells were cultured in the presence and in the absence of IL-1 β and of CSE (10%) (n = 3) (see materials and methods for details). **A**) Expression of HBD2 m-RNA in 16-HBE by real time PCR. GAPDH gene expression was used as endogenous control for normalization. Relative quantitation of mRNA was carried out with comparative CT method. (mean±SD). * p<0.05 versus baseline. ** p<0.05 versus IL-1 beta. **B**) Representative experiment (one out of three experiments) showing the expression of HBD2 protein in 16-HBE by flow cytometry. The expression of HBD2 is expressed as percentage of HBD2 positive cells. **C**) Evaluation of p-IkBa or t-IkBa by western blot analysis. Membranes were then stripped and incubated with goat polyclonal anti–ß-actin. Representative western blot analysis (one out of three experiments). Lane1 = baseline; lane 2 = CSE 10%;lane 3 = IL1 beta; lane 4 = CSE+IL1 beta. **D**) ChiP assay using anti-NFkB antibody and PCR using primers (forward 5′-GGTGTGAATGGAAGGAACTCA-3′ reverse 5′-TTCAGCTCCTGGGGATGATAC-3′) spanning the promoter region of HBD2 gene were performed (see Materials and Methods for details) One out of two experiments is shown. Lane 1 = DNA marker; Lane 2 = baseline; lane 3 = CSE 10%; lane 4 = IL1 β; lane 5 = CSE+IL1 β.

To better explore the role of cigarette smoke exposure, HBD2 expression at both mRNA (figure 7 A) and protein level (figure 7 B) was evaluated in IL1 beta and CSE stimulated bronchial epithelial cells (16-HBE). CSE did not modify the constitutive expression of HBD2. IL1 beta induced the HBD2 expression and CSE significantly counteracted this IL-1 β mediated effect further supporting the negative effect of cigarette smoke in the expression of HBD2 by bronchial epithelial cells. The mechanisms underlining the inhibitory effects of CSE in HBD2 induction were further explored. In bronchial epithelial cells, IL-1 beta increased the expression of pIKBa leading to an increased activation of NFkB pathway and CSE negatively interfered with this phenomenon (figure 7 C).

Furthermore, we carried out ChIP assays with antibodies specific to NFkB (figure 7 D) and the results of these experiments showed that NFkB is detected on the promoter region of HBD2 in bronchial epithelial cells after incubation with IL-1 β and this phenomenon was reverted by the presence of CSE.

## Discussion

COPD is a poorly understood and slowly evolving disease where significant pathological anathomical changes are already present at the diagnosis. Detailed inflammatory profiles of disease phenotypes are now emerging in COPD.

This study demonstrates for the first time that an over-expression of TLR4 is present in the epithelium of both central and distal airways of s-COPD. HBD2 epithelial expression is reduced in the epithelium of central airways while it is increased in the epithelium of distal airways of s-COPD and this marker correlates with airflow obstruction and with the packs/year of smoking. The reduced expression of HBD2 in the epithelium of central airways is due to the negative effect of CSE in NFkB pathway activation.

In COPD, the predominant pathology is present in peripheral airways and lung parenchyma [12]. To what extent central airways may mirror events occurring in distal lung is uncertain. Neutrophils are more numerous in the proximal bronchial tree and macrophages are predominantly present in distal airways [22]. The inflammatory processes promote the structural and functional changes associated with chronic bronchitis in the larger bronchi [23] while in the smaller bronchi and bronchioles, they cause the occlusion of the lumen by mucus, thickening of the walls, and narrowing of the lumen [12]. This study was designed to understand whether innate immunity response mechanisms are differently altered at different levels of the bronchial tree in smokers and in COPD patients (current smokers and ex-smokers) with stable disease.

Innate immunity relies on pattern recognition receptors that recognize structures common to many microorganisms and endogenous ligands such as heat shock proteins. A study from Pons [24] showed that TLR-2 is up-regulated in peripheral blood monocytes harvested from COPD patients, either when clinically stable or when exacerbated. Droemann et al. [25] reported that alveolar macrophages from stable COPD patients and smokers express less TLR-2 than never smokers. We demonstrate here for the first time that TLR4 expression is increased in central and distal airway epithelium in both smokers and s-COPD. These findings strongly suggest that the expression of TLRs may be differently modified in different cell compartments (immunocompetent cells or airway epithelial cells) of the bronchial tree. Furthermore, the increased expression of TLR4 in the epithelium of central airways in smokers and in s-COPD is consistent with the results of a previous *in vitro* study published by our group showing that CSE increase the expression of TLR4 in a bronchial epithelial cell line [11].

TLRs establish the inflammatory setting in response to infections or tissue damage and provides a low-grade activation of the innate immune system for day-to-day lung structure stability [25]. High grade activation of TLR signalling leading to increased production of cytokines and reactive oxidant contributes to experimental emphysema [26].

CSE in mice induce airway neutrophilia via activation of TLR4 signalling [27] and in bronchial epithelial cells, *in vitro*, orientate the activation of TLR4 toward an increased IL-8 release and a reduced IP-10 release leading to an increased neutrophil chemotaxis and to a reduced lymphocyte chemotaxis thus altering the balance between innate and adaptative responses [11]. This unbalance may amplify lung inflammation since lung inflammation can be excessive when the adaptive pulmonary immune responses are inappropriate [28]. TLR4 activation by external agents is mainly due to gram negative bacteria and is also finalized to the release of antimicrobial peptides including HBD2, a molecule with a potent effect against gram negative bacteria [20]. Respiratory epithelial cells require TLR4 for the induction of HBD2 by LPS [29]. HBD2, mainly present in structural epithelial cells, exerts specific chemotactic activity for neutrophils [30] and may amplify TLR responses acting as an endogenous TLR ligand [31]. We show here that HBD2 is reduced in central airways of s-COPD patients when compared to smoker subjects and COPD who stopped to smoke and correlates with the degree of airway obstruction assessed by the reduction in FEV1/FVC ratio that, as previously reported [32], is a good spirometric parameter to represent airflow limitation. Moreover, HBD2 expression in central airways inversely correlates with pack/years of smoking, strongly suggesting that cigarette smoke exposure crucially negatively affects the expression of HBD2 in COPD patients. In this regard, it has been recently demonstrated that cigarette smoke extracts reduce the expression of HBD2 in primary bronchial epithelial cells from smokers and from COPD patients [9].Our in vitro experiments showing that the exposure of CSE in bronchial epithelial cells blocks the induction of HBD2 mRNA generated by exposure to IL-1 β, a cytokine with a crucial role in the inflammation of COPD, confirm and extend these observation providing some explanation on the mechanisms that contribute to this phenomenon. Cigarette smoke interferes with the innate host defence system by increasing mucus production, reducing mucociliary clearance, disrupting the epithelial barrier and stimulating the migration of inflammatory and immune cells [28]. In addition, the exposure of airway epithelium to smoke blocks the LPS-induced activation of NFkB pathway [11] [33], a signal pathway with a crucial role in the HBD2 synthesis. Accordingly, it has been previously described that the exposure of airway epithelium to smoke inhibits the HBD2 induction by bacteria [34]. To further support these data, in the present study, we demonstrate that CSE inhibits IL-1 β induced NFkB pathway activation and in turn negatively interferes with the interaction between NFkB and the promoter region of HBD2. Subjects with reduced HBD2 gene copies are predisposed to Crohn's disease [35] and here, a reduced HBD2 expression in the epithelium of central airways is present in s-COPD further supporting the concept that the de-regulation of the HBD2 expression in a specific compartment of the bronchial tree may contribute to the disease development. Additional mechanisms may account for the reduced HBD2 expression in central airways of current smoker COPD. HBD2 synthesis may be promoted by leptin [36] and in epithelial cells of bronchial biopsies, the expression of leptin and its receptors is reduced in mild-to-severe COPD patients [37].

Since not all smokers develop COPD [38], the reduced expression of HBD2 in central airways might identify smokers susceptible to develop COPD. Further studies are needed to validate this hypothesis. Moreover, decreased HBD2 together with an increased TLR4 expression in central airway epithelium may suggest an impairment in the activation of innate responses at this level that in turn may favour the microbial invasion to distal airways and to the parenchyma. Physiologically, the distal airways are sterile while the airways of COPD patients are chronically colonized by potential respiratory pathogens [39]. Chronic bacterial colonization together to an oxidant/antioxidant unbalance can stimulate the host immune system and cause a chronic airway inflammation [40] that in turn may promote the tissue damage observed in distal airways and lung parenchyma of COPD patients. Bronchiolar inflammation correlates with functional impairment and temporally precedes emphysema [22]. Our findings that in distal airways of s-COPD both HBD2 and TLR4 epithelial expression are increased support the concept that an increased activation of innate immunity responses may occur at this level. In the distal airways of smokers with COPD and acute respiratory failure high levels of HBD2 increase neutrophil survival [40] thus contributing to amplify the inflammatory responses which, in turn, promote the occlusion of the lumen by mucus, thickening of the walls, and narrowing. In addition, ex-s-COPD have an increased epithelial HBD2 expression in central airways further supporting the concept that smoking cessation may alter the inflammatory profile of the airway epithelial cells. Ex-smokers with COPD have significantly less epithelial squamous cell metaplasia, proliferating cell numbers, and show a trend towards reduced goblet cell area than current smokers with COPD [41].

In conclusion, this study demonstrates that although an over-expression of TLR4 is present in central and in distal airways of s-COPD and of S, HBD2 is reduced in central airways but not in distal airways of s-COPD and correlates with the degree of airflow obstruction and with smoking history.
